# DOCK8 inhibits the immune function of neutrophils in sepsis by regulating aerobic glycolysis

**DOI:** 10.1002/iid3.965

**Published:** 2023-08-28

**Authors:** Hongjun Zhu, Junlong Xu, Ke Li, Miaomiao Chen, Yueming Wu, Xian Zhang, Hua Chen, Deyuan Chen

**Affiliations:** ^1^ Clinical Laboratory, The Sixth Affiliated Hospital of Wenzhou Medical University Lishui People's Hospital Lishui City China; ^2^ Department of Critical Care Medicine, The Sixth Affiliated Hospital of Wenzhou Medical University Lishui People's Hospital Lishui City China

**Keywords:** aerobic glycolysis, DOCK8, immune function, neutrophils, sepsis

## Abstract

**Introduction:**

This study endeavored to investigate the role of DOCK8 in modulating the immune function triggered by sepsis.

**Methods:**

Expression of DOCK8 in the whole blood of sepsis patients and its enrichment pathways were assayed by bioinformatics. Pearson analysis was used to predict the relationship between glycolytic signaling pathway and its relevance to neutrophil function in sepsis. A sepsis mouse model was then built by performing cecal ligation and puncture treatment on male mice. Neutrophils were isolated, and their purity was tested by flow cytometry. Neutrophils were then stimulated by lipopolysaccharide to build a sepsis cell model. Next, quantitative reverse transcription polymerase chain reaction and CCK‐8 were applied to test the expression of DOCK8 and cell viability, western blot to assay the expression of HK‐2, PKM2, and LDHA proteins, ELISA to measure the concentrations of TNF‐α, IL‐1β, and IL‐6, Transwell to detect the chemotaxis of neutrophils and flow cytometry to detect the phagocytic activity of neutrophils. Finally, in different treatment groups, we used Seahorse XF 96 to analyze the extracellular acidification rate (ECAR) of sepsis cells and used enzyme‐linked immunosorbent assay to detect the contents of pyruvic acid, lactic acid, and ATP in sepsis cells.

**Results:**

DOCK8 was downregulated in sepsis blood and activated neutrophils. Aerobic glycolysis was positively correlated with sepsis. Activated neutrophils promoted the expression of inflammatory factors TNF‐α, IL‐1β, and IL‐6. Low expression of DOCK8 facilitated the proliferation, chemotaxis, and phagocytic activity of sepsis cells and promoted the expression of inflammatory factors. Bioinformatics analysis revealed that DOCK8 was enriched in the glycolytic signaling pathway. Low expression of DOCK8 induced ECAR, promoted the protein expression of HK‐2, PKM2 and LDHA, and favored the increase of pyruvate, lactate, and ATP contents. While 2‐DG treatment could restore these effects.

**Conclusion:**

DOCK8 may inhibit sepsis‐induced neutrophil immune function by regulating aerobic glycolysis and causing excessive inflammation, which helps to explore potential therapeutic targets.

## Graphical abstract

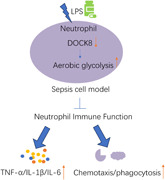

## 1 INTRODUCTION

Sepsis, a life‐threatening syndrome, arises from a dysregulated host response to infection leading to organ dysfunction.^1^ Currently, sepsis has become the most common cause of death in the intensive care unit.^2^ During sepsis, the immune function of the host is dysregulated, leading to inadequate clearance of pathogenic bacteria, which results in aggravated multiple organ dysfunction and, ultimately, the death of septic patients.^3^, ^4^ Given the intricate nature of sepsis pathogenesis, the pathological and physiological mechanisms underlying sepsis remain incompletely understood, and there are still no specific diagnostic methods and effective treatments available. Understanding the pathological and physiological mechanisms of sepsis is crucial for precise treatment and reducing patient mortality. Therefore, this study was devised to explore the potential pathogenesis of sepsis and identify potential therapeutic targets.

Neutrophils are myeloid white blood cells and the most important component of the innate immune system.^5^ When the host is invaded by pathogenic bacteria, neutrophils are recruited to the site of infection to eliminate the pathogen.^5^ In the early stage of sepsis, neutrophils are the essential first line of defense for clearing pathogenic bacteria.^6^ In sepsis, neutrophils contact and kill microorganisms by various mechanisms, including chemotaxis, phagocytosis, reactive oxygen species release, production and release of granule enzymes and cytokine, and formation of neutrophil extracellular traps (NETs), and regulate immune function through interactions with other immune cells.^7^ For instance, Jeroen et al.^8^ found that during sepsis, IFN‐γ can induce high expression of PD‐L1 in neutrophils, which negatively regulates lymphocytes through inhibiting their proliferation, activation, and inflammatory cytokine release, and inducing lymphocyte apoptosis via the PD‐L1 signaling pathway. Guang et al.^9^ found that the chemotactic factor CD177 or cytokines produced by neutrophils are involved in coordinating the intestinal mucosal immune function and negatively regulate IBD. However, the immune regulatory pathways of neutrophils during this condition remain incompletely characterized.

DOCK8 is a well‐studied member of the DOCK protein family, which is an atypical guanine nucleotide exchange factor (GEF).^10^ DOCK8 is highly expressed in both B and T cells, and much research has focused on its role in the immune system.^11^ For example, the expression of DOCK8 in regulatory T cells (Tregs) limits contact hypersensitivity by fostering the stability and adaptability of Tregs in inflamed skin.^12^ Additionally, the decreased expression level of DOCK8 and the abnormal signaling pathway driven by DOCK8 in autoimmune uveitis can promote the process of auto‐reactive inflammation.^13^ Nevertheless, the precise role of DOCK8 in regulating sepsis neutrophil immune function remains poorly understood. Consequently, further investigation is needed to delineate the function of DOCK8 in mice and humans, as well as to differentiate its unique effects on immunity and diseases from those of other DOCK proteins. Hence, this study aims to probe into the role of DOCK8 in the pathogenesis of sepsis and its involvement in immune function, with the aim of providing new insights into the disease mechanism.

In this study, we first constructed cecal ligation and puncture (CLP) mouse model and a sepsis cell model to analyze the expression of DOCK8 in sepsis and sepsis Neutrophil, and clarified the impact of DOCK8 on sepsis Neutrophil immune function. Mechanistically, DOCK8 was found to be downregulated in sepsis and sepsis neutrophil, and enriched in the aerobic glycolytic pathway. In the rescue assay using 2‐DG, we discovered that DOCK8 regulated aerobic glycolysis, thereby inhibiting the immune function of sepsis neutrophils. These findings had the potential to provide novel and effective therapeutic targets for improving the treatment of sepsis.

## 2 MATERIALS AND METHODS

### 2.1 Bioinformatics analysis

The gene expression data of whole blood samples from critically ill sepsis patients (GSE134347) were downloaded from Gene Expression Omnibus (GEO) database, which included 83 normal samples and 156 severe sepsis samples. Differential analysis of mRNA was performed using the “limma” package to obtain differentially expressed mRNAs from the normal group and sepsis group (|FC|> 1, *p*_adj_ < .05). Based on the literature review, we selected the target gene for our study and used the Wilcoxon test to determine the expression differences of the target gene between normal and severe sepsis samples. Pearson analysis was used to predict the relationship between the glycolytic signaling pathway and its relevance to neutrophil function in sepsis. The literature citation and Gene Set Enrichment Analysis were conducted.^14^

### 2.2 CLP mouse model

Male C57BL/6J mice, aged 8‐12 weeks and weighing 20–25 g, were obtained from GemPharmatech Experimental Animal Corporation (Nanjing, China). The mice were acclimated to a specific pathogen‐free environment for 1 week and were maintained under a 12‐h light/dark cycle with ad libitum access to food and water. All mice were randomly assigned to four groups (*n* = 5 per group). After anesthetizing the mice with isoflurane, the cecum was exposed through a midline abdominal incision. The mid‐segment of the cecum was ligated with 5.0 silk thread and punctured twice with a 20‐gauge needle on the same side. The cecum was then returned to the abdominal cavity and sutured. All sham‐operated mice received the same surgical procedure except for ligation and puncture. Postoperative analgesia was achieved by subcutaneous injection of buprenorphine (0.05 mg/kg). At 24 h after surgery, mice were euthanized by cardiac puncture under isoflurane anesthesia, and blood samples were collected for further experiments. All procedures involving live mice were performed following the current guidelines and protocols approved by the Animal Care and Use Committee of Lishui University.^15^

### 2.3 Neutrophil separation

Neutrophils were separated using a mouse Neutrophil isolation kit, followed by fluorescence‐activated cell sorting analysis using antibodies against Ly‐6G/Ly‐6C (Thermo) and CD11b (Thermo) to assess the purity of the isolated mouse neutrophils.^16^

### 2.4 Lipopolysaccharide (LPS)‐stimulated sepsis cell model construction

Purified mouse neutrophils were stimulated with LPS (Sigma) at a concentration of 100 ng/mL in RPMI‐1640 medium for 12 h to simulate the activation of neutrophils in sepsis.^17^

### 2.5 Cell transfection and rescue experiment

RiboBio provided us with sh‐DOCK8, oe‐DOCK8, and their negative controls. Following the instructions of the kit, we transfected the above plasmids into neutrophils from sepsis using Lipofectamine 2000 (Invitrogen) and conducted the next experiment 24 h after cultivation. Based on the grouping of transfected sh‐DOCK8 and its controls, 2‐DG was added to set up a rescue assay, with the groups of sh‐NC+PBS, sh‐DOCK8+PBS, and sh‐DOCK8+2‐DG.

### 2.6 Quantitative reverse transcription polymerase chain reaction (qRT‐PCR)

Total RNA was extracted using a Trizol reagent. For each sample, 0.5 μg of RNA was reverse‐transcribed utilizing a PrimeScript RT kit (TAKARA Bio). Real‐time fluorescence quantitative PCR analysis was performed utilizing Power SYBR Green PCR pre‐mix (Roche Diagnostics), with β‐Actin as the control.^18^ The relative RNA levels were analyzed using the 2^−ΔΔCt^ method. The primer sequences are shown in Table 1.

**Table 1:** Primer sequences.

Gene	Sequences	
DOCK8	Forward Primer	5′‐TGGCCTTCACACCCAAAGAA‐3′
	Reverse Primer	5′‐GAACACAGTCTCTGACGTGAGG‐3′
β‐Actin	Forward Primer	5′‐ACCGTGAGAAGATGACCCAGA‐3′
	Reverse Primer	5′‐AGAGGCGTACAGGGACAGCA‐3′

### 2.7 Western blot (WB)

The method as followed the previous article.^19^ Cells were gathered and treated with lysis buffer. Equal amounts of total proteins (20 μg) were separated by sodium dodecyl sulfate‐polyacrylamide gel electrophoresis and transferred onto a polyvinylidene fluoride membrane (EMD Millipore). The membrane was blocked with 5% skimmed milk and incubated with the corresponding primary antibodies overnight at 4°C. After washing with 1× TBST three times, the membrane was incubated with the horseradish peroxidase‐conjugated secondary antibody (goat anti‐rabbit IgG) for 1 h at room temperature. Following three washes with 1× TBST, the immunoreactivity was detected using an enhanced chemiluminescence method and captured using a chemiluminescence imaging system. The primary antibodies used were Anti‐HK‐2, Anti‐PKM2, Anti‐LDHA, and Anti‐β‐Actin (all rabbit anti‐human), and the secondary antibody was goat anti‐rabbit IgG. All of the antibodies were purchased from Abcam.

### 2.8 Cell Counting Kit‐8 (CCK‐8)

CCK‐8 assay was utilized to determine cell viability. Cells were seeded in 96‐well plates and pre‐incubated for 0 h, 24 h, 48 h, and 72 h under 37°C with 5% CO_2_. Then, 10 μL of CCK‐8 reagent was added to each well and incubated for 4 h at 37°C and 5% CO_2_. The optical density (OD) at 450 nm of each well was measured utilizing an enzyme‐linked immunosorbent assay (ELISA) reader. All reactions were repeated three times.^20^

### 2.9 ELISA

Levels of inflammatory cytokines (tumor necrosis factor α [TNF‐α], interleukin [IL]‐1β, and IL‐6) were assayed using an ELISA assay kit (Abcam). LPS‐stimulated neutrophils were added to a 96‐well microtiter plate containing specific immobilized antibodies that could bind TNF‐α, IL‐1β, or IL‐6. After washing with a washing buffer to remove unbound substances and antibodies, a stop solution was added to terminate the color development, and the OD value at 540 nm was measured in an ELISA plate reader. The concentrations were calculated from the standard curve and multiplied by the dilution factor to obtain the results.^21^

### 2.10 Neutrophil chemotaxis assay

We measured the neutrophil chemotaxis in a 96‐well chemotaxis chamber. First, fMLP (50 nM) was filled in the wells, and neutrophil (5 × 10^4^) was suspended in RPMI‐1640 medium. A 3.0 μm filter membrane was placed in the loading well, and then 25 μL of Neutrophil (2 × 10^6^/mL) was added to the filter membrane. The chamber was incubated at 37°C with 5% CO_2_ for 1 h. After washing the wells and the filter membrane with 25 μL of RPMI‐1640 medium, the plate and the attached filter membrane were centrifuged at 350 *g* for 10 min to remove the migrated cells underneath the filter. The filter was removed, and the neutrophils in the chemotaxis wells were suspended and counted using a hemocytometer.^22^

### 2.11 Neutrophil phagocytosis assay

The assay was conducted utilizing flow cytometry. 100 μL of cells with a concentration of 1 × 10^4^ cells/μL were mixed with 10 μL of FluoSpheres fluorescent microspheres (1 × 10^10^ microspheres/mL) (Invitrogen) and incubated at 37°C for 40 min. During this process, neutrophils engulfed the microspheres. After being washed five times with phosphate buffered saline (PBS) to eliminate free particles, the cells were resuspended in 1 mL of PBS, added the CD11b‐FITC and Ly‐6G‐Ly‐6C‐PE. Then subjected to flow cytometry analysis using a flow cytometer (Becton Dickinson).^22^ The negative/positive control group was used to gating strategy. Negative control group means only added the CD11b‐FITC antibody, positive control group means only added the Ly‐6G‐Ly‐6C‐PE antibody.

### 2.12 Extracellular acidification rate (ECAR) measurement

The XF 96 Cell Energy Metabolism Analyzer (Seahorse Bioscience, USA) was used for real‐time analysis of ECAR. Briefly, the sensors were immersed in a calibration solution and placed in a CO_2_‐free incubator overnight. Neutrophils (5 × 10^4^ cells/well) were then seeded into the XF 96 cell culture plate and incubated overnight. ECAR was measured after injection of glucose (10 mM), oligomycin (1 μM), and 2‐DG (100 mM) into each well.^23^

### 2.13 Measurement of acetate, lactate, and ATP

Lactate assay kit (Solarbio), acetate assay kit (Solarbio), and ATP colorimetric/fluorescent assay kit (Solarbio) were utilized to assess the levels of lactate accumulation, acetate, and ATP in neutrophils.^24^

### 2.14 Data analysis

All experiments were repeated three times. The data were expressed as mean ± SD and analyzed using GraphPad 8.0 software. *T*‐test was employed to analyze differences between the two groups, whereas one‐way analysis of variance was used for comparison among multiple groups. The *p* < .05 indicates statistical significance, *p* < .01 indicates significant difference, and *p* < .001 indicates extremely significant difference.

## 3 RESULTS

### 3.1 Low expression of DOCK8 in sepsis

**Figure 1:**
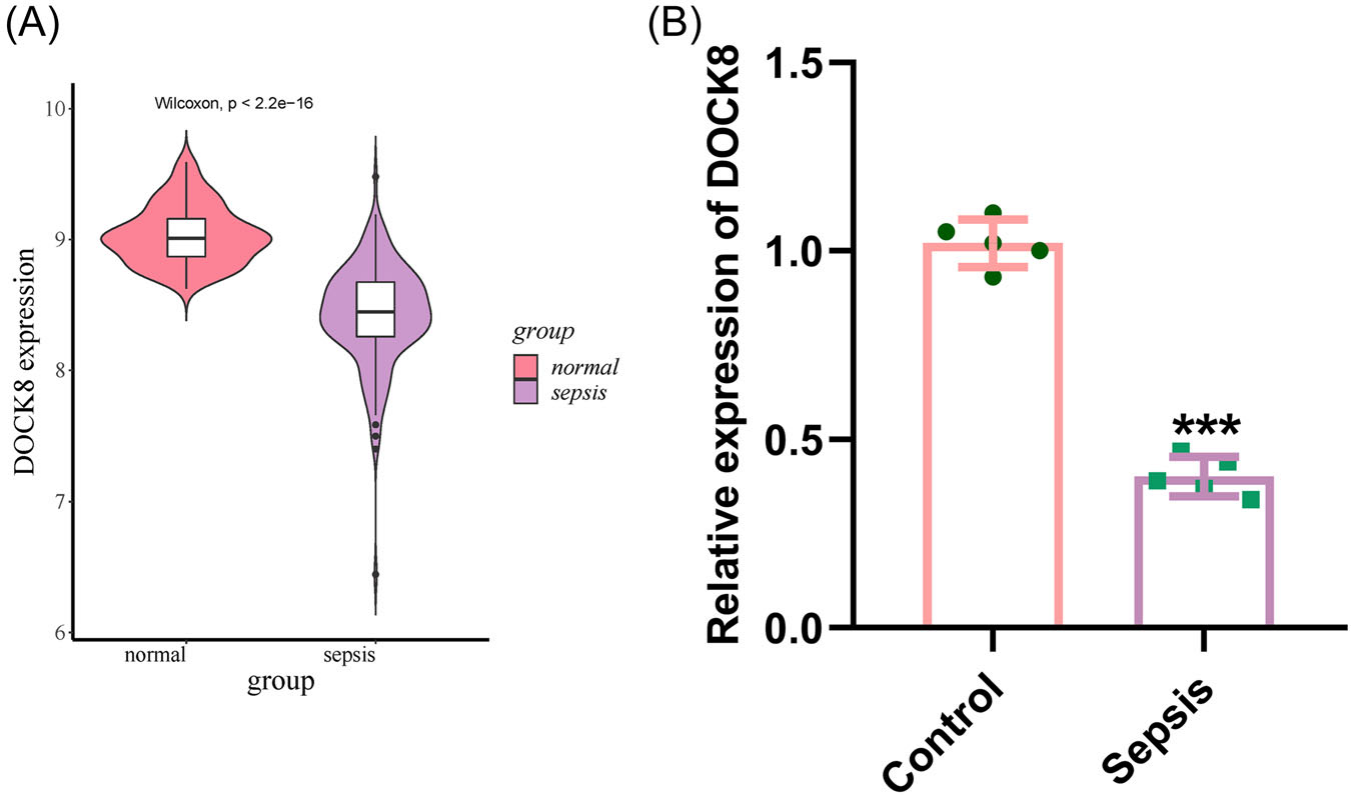
Low expression of DOCK8 in Sepsis. (A) Bioinformatics analysis of DOCK8 expression in sepsis blood; (B) DOCK8 expression in sepsis mouse blood. All experiments were repeated three times. ***p < .001.

Bioinformatics analysis of gene expression data from the whole blood of critically ill sepsis patients in the GEO database (GSE134347, which included 83 normal samples and 156 severe sepsis samples) revealed low expression of DOCK8 in sepsis patients (*p* < 2.2e−16, Figure 1A). To investigate the potential role of DOCK8 in sepsis, we established a CLP mouse model. qRT‐PCR analysis showed that the expression of DOCK8 was significantly lower in the CLP mouse model compared to the sham surgery group (*p* < .001, Figure 1B), indicating a low expression of DOCK8 in sepsis.

### 3.2 Reduced expression of DOCK8 in neutrophils during sepsis

**Figure 2:**
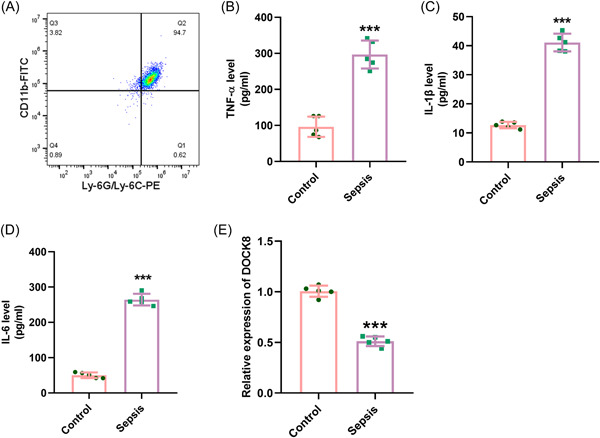
Reduced expression of DOCK8 in neutrophils during sepsis. (A) Flow cytometry analysis of neutrophil purity; (B–D) ELISA analysis of TNF‐α, IL‐1β, and IL‐6 concentrations in cells; (E) qRT‐PCR analysis of DOCK8 expression. All experiments were repeated three times. ***p < .001. ELISA, enzyme‐linked immunosorbent assay; IL, interleukin; qRT‐PCR, quantitative reverse transcription polymerase chain reaction; TNF‐α, tumor necrosis factor α.

During the early stage of sepsis, neutrophils act as the first line of defense and are crucial for eliminating pathogenic bacteria.^6^, ^21^ To investigate the expression of DOCK8 in neutrophils during sepsis, we isolated neutrophils from the blood of sepsis mice and assessed their purity. The results showed that the purity of neutrophils was >93%, indicating successful isolation (Figure 2A). To confirm whether the inflammatory response of neutrophils was activated, we examined the expression of inflammatory cytokines TNF‐α, IL‐1β, and IL‐6 in neutrophils. It was found that in the CLP mouse model, the expression of inflammatory cytokines was markedly increased compared to the sham group (*p* < .001, Figure 2B–D). Additionally, qRT‐PCR analysis demonstrated a significant decrease in DOCK8 expression in activated neutrophils (Figure 2E), suggesting a reduced expression of DOCK8 in neutrophils during sepsis.

### 3.3 DOCK8 inhibition of neutrophil immune function in sepsis

**Figure 3:**
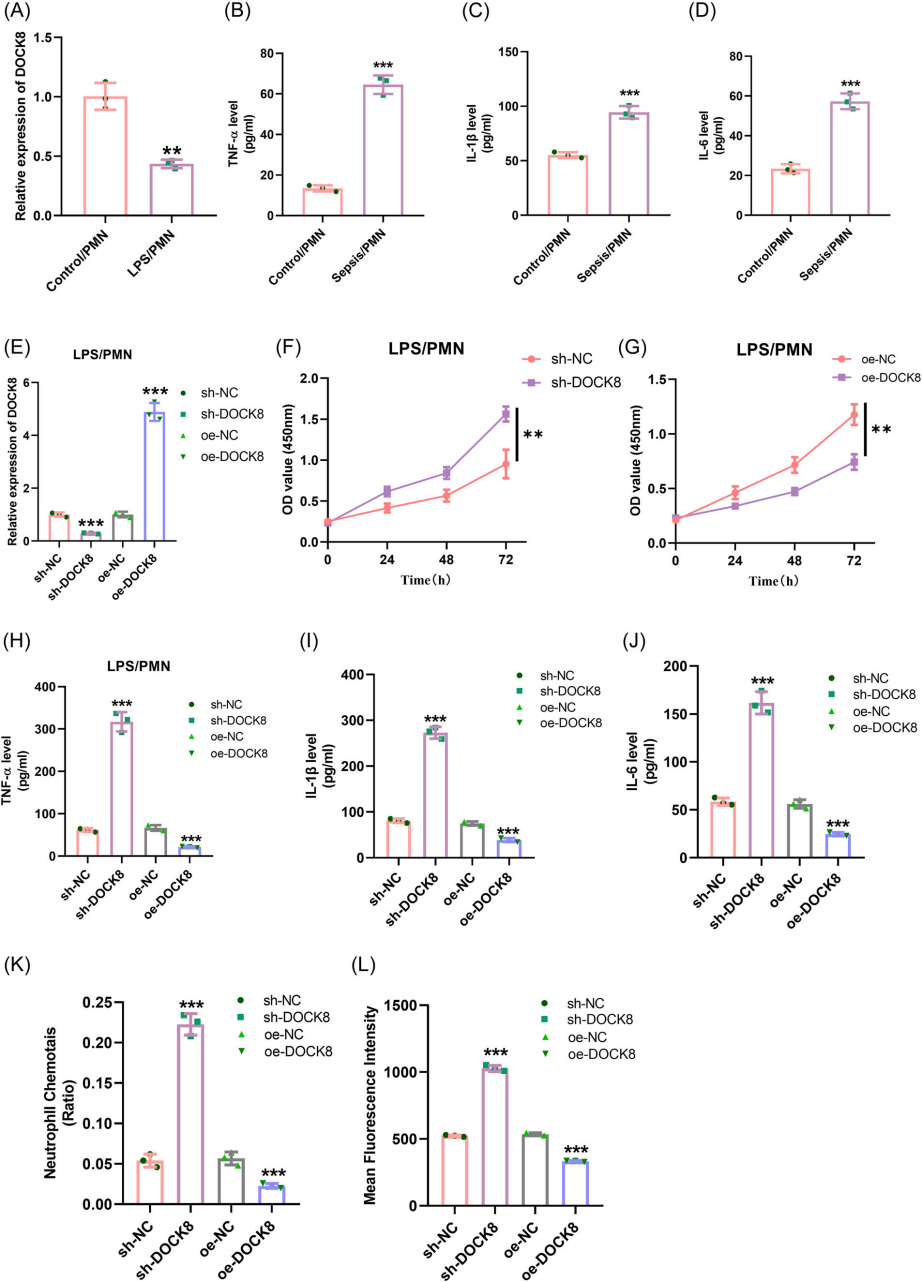
DOCK8 inhibition of neutrophil immune function in sepsis. (A) qRT‐PCR detection of DOCK8 expression; (B–D) ELISA detection of TNF‐α, IL‐1β, and IL‐6 concentrations in cells; (E) qRT‐PCR detection of transfection efficiency; (F–G) CCK‐8 detection of cell viability; (H–J) ELISA detection of TNF‐α, IL‐1β, and IL‐6 concentrations in cells; (K) transwell assay for neutrophil chemotaxis; (L) elow cytometry detection of phagocytosis; All experiments were repeated three times. **p < .01, ***p < .001. CCK‐8, Counting Kit‐8; ELISA, enzyme‐linked immunosorbent assay; IL, interleukin; qRT‐PCR, quantitative reverse transcription polymerase chain reaction; TNF‐α, tumor necrosis factor α.

To further confirm our findings, we stimulated human neutrophils with LPS to construct a sepsis cell model and simulate the activation of neutrophils in sepsis. We performed qRT‐PCR to detect the expression of DOCK8 and found that DOCK8 was significantly downregulated in the sepsis cell model (*p* < .01, Figure 3A). We also performed ELISA to assay the expression of inflammatory cytokines, and results revealed that the concentrations of TNF‐α, IL‐1β, and IL‐6 were notably increased in the sepsis cell model (*p* < .001, Figure 3B–D).

To probe into the mechanism of neutrophil immune metabolism in sepsis, we knocked down or overexpressed DOCK8 and its negative control in the sepsis cell model, and then measured the transfection efficiency using qRT‐PCR. Our findings showed that the expression of DOCK8 was tellingly downregulated in the sepsis cell model after knocking down DOCK8, and significantly upregulated after overexpressing DOCK8 (*p* < .001, Figure 3E). We also used CCK‐8 to detect cell viability and found that knocking down DOCK8 significantly promoted the activity of the sepsis cell model, while overexpressing DOCK8 significantly inhibited its activity (*p* < .01, Figure 3F,G). Furthermore, we performed ELISA to assay the expression of inflammatory cytokines and found that knocking down DOCK8 fostered the expression of inflammatory cytokines while overexpressing DOCK8 inhibited their expression (*p* < .001, Figure 3H–J). We also used a Transwell chemotaxis assay and phagocytosis assay to detect the immune function of the sepsis cell model and found that knocking down DOCK8 significantly increased the levels of chemotaxis and phagocytosis, while overexpressing DOCK8 significantly decreased these levels (*p* < .001, Figure 3K,L). These findings illustrated that DOCK8 inhibited the immune function of neutrophils in sepsis.

### 3.4 DOCK8 inhibits neutrophil immune function in sepsis via aerobic glycolysis

**Figure 4:**
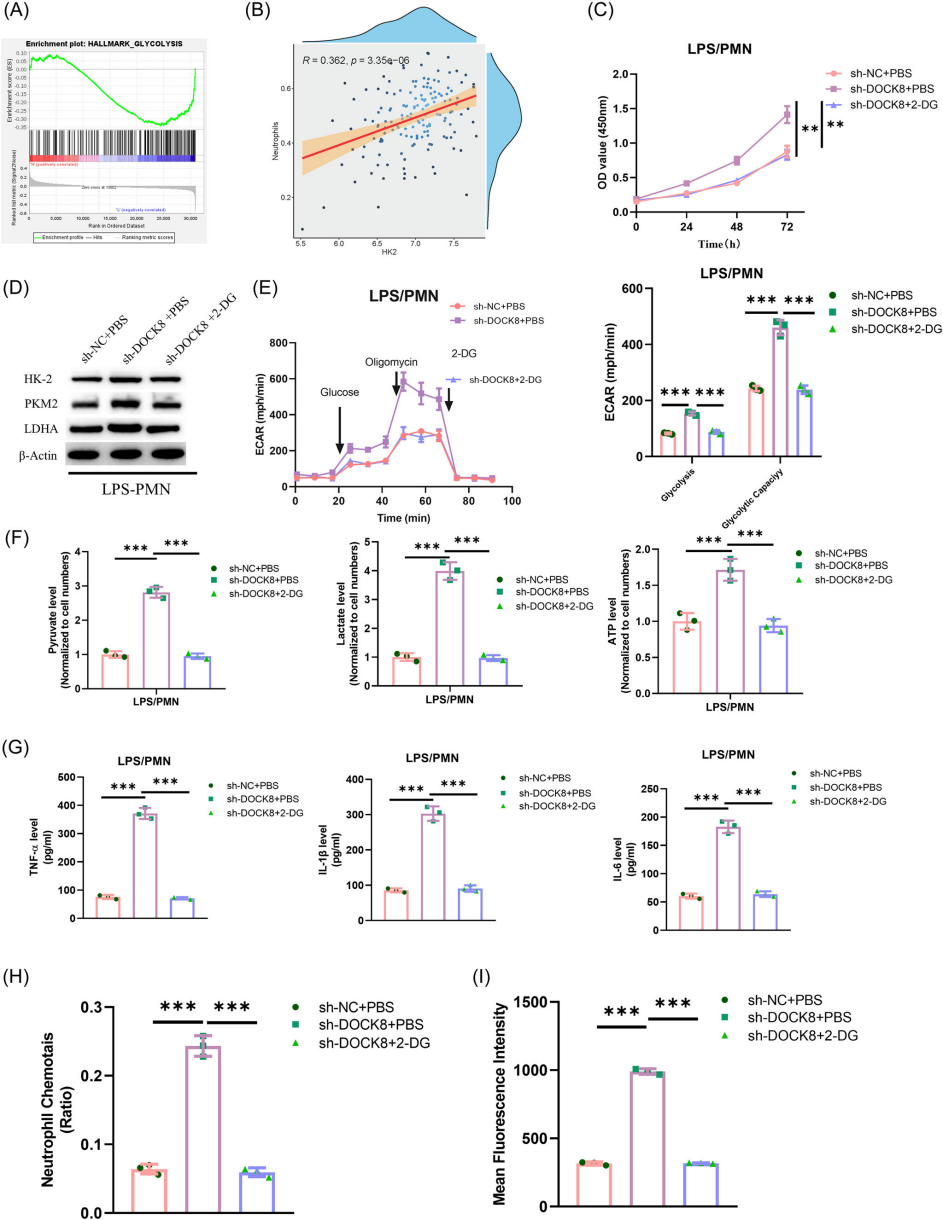
DOCK8 suppresses the immune function of neutrophils in sepsis through aerobic glycolysis inhibition. (A) Enrichment analysis of DOCK8 pathway; (B) Pearson analysis was used to predict the relationship between glycolytic signaling pathway and its relevance to neutrophil function in sepsis; (C) CCK‐8 assay for cell viability; (D) western blot analysis of the expression levels of specific genes (HK2, PKM2, and LDHA) related to glycolytic metabolism pathway; (E) seahorse XF 96 analyses of ECAR; (F) detection of the levels of pyruvate, lactate, and ATP in sepsis cells of each treatment group; (G) ELISA detection of TNF‐α, IL‐1β, and IL‐6 concentrations in cells; (H) transwell assay was utilized to measure the chemotactic effect of Neutrophils; and (I) flow cytometry analysis of phagocytic function. All experiments were repeated three times. **p < .01, ***p < .001. CCK‐8, Counting Kit‐8; ELISA, enzyme‐linked immunosorbent assay; IL, interleukin; qRT‐PCR, quantitative reverse transcription polymerase chain reaction; TNF‐α, tumor necrosis factor α.

To further elucidate the potential mechanism of DOCK8 in affecting Neutrophil immune function in sepsis, we analyzed the enrichment pathways of DOCK8 through bioinformatics and found that DOCK8 was enriched in the glycolytic signaling pathway (Figure 4A). Some studies have reported that aerobic glycolysis fosters the immune function of neutrophils in sepsis.^22^ Therefore, we hypothesized that DOCK8 may regulate aerobic glycolysis to inhibit the immune function of neutrophils in sepsis. Meanwhile, Pearson analysis showed that glycolysis was positively correlated with sepsis (*p* < .001, Figure 4B). To verify it, we conducted a rescue assay using the glycolysis inhibitor 2‐DG. First, we established cell groups based on a sepsis cell model: sh‐NC + PBS, sh‐DOCK8 + PBS, and sh‐DOCK8 + 2‐DG. CCK‐8 results illustrated that knocking down DOCK8 notably promoted the activity of neutrophils in sepsis, and treatment with 2‐DG could restore this effect (*p* < .01, Figure 4C). WB was performed to detect the expression of specific genes related to the glycolytic metabolic pathway, with outcomes showing that knocking down DOCK8 tellingly promoted the expression of HK2, PKM2, and LDHA. However, treatment with 2‐DG could restore the promotion of knocking down DOCK8 on the expression of HK2, PKM2, and LDHA (Figure 4D). Seahorse XF 96 analyses of ECAR showed that knocking down DOCK8 significantly increased the ECAR of sepsis neutrophils. However, treatment with 2‐DG could restore these effects (*p* < .001, Figure 4E). Further detection of the levels of pyruvic acid, lactic acid, and ATP in sepsis cells of each treatment group showed that knocking down DOCK8 significantly increased the levels of pyruvic acid, lactic acid, and ATP. However, treatment with 2‐DG could restore these effects (*p* < .001, Figure 4F). ELISA analysis of the expression of inflammatory factors revealed that treatment with 2‐DG could restore the promotion of knocking down DOCK8 on the expression of inflammatory factors (*p* < .001, Figure 4G). Subsequently, Transwell chemotaxis experiments and phagocytosis experiments were performed to test the immune function, and the results showed that knocking down DOCK8 significantly increased the levels of chemotaxis and phagocytosis in the sepsis cell model. However, treatment with 2‐DG could restore the promoting effect of knocking down DOCK8 (*p* < .001, Figure 4H,I). In conclusion, DOCK8 inhibited the immune function of neutrophils in sepsis via aerobic glycolysis.

## 4 DISCUSSION

Sepsis is one of the most common complications in patients with severe trauma, severe burns, shock, infection, or major surgery, characterized by fever, chills, respiratory alkalosis, severe hypothermia, or edema.^25^ Late‐stage sepsis triggers immune suppression, which leads to sustained or recurrent infection.^26^ The incidence of sepsis is estimated to be 0.3%, with a mortality rate of 20%–40%.^27^ Suppressing the high inflammatory response in the early stages of sepsis can reduce damage to immune cells, inhibit immune cell apoptosis, and is a key step in preventing and reducing sepsis‐induced immune suppression.^28^ Hence, it is of paramount importance to explore effective strategies to alleviate the early inflammatory responses in sepsis for its optimal management.

The early inflammatory response in sepsis is primarily initiated by innate immune cells of the immune system, including neutrophils, monocytes, and macrophages, which are capable of producing numerous inflammatory cytokines.^29^ Neutrophils feature in the sepsis‐induced pathological physiology and immune dysfunction, and serve as the initial important line of defense against infection in the host.^30^ Overactivation of neutrophils, as an important pathological mechanism of organ damage in sepsis, is detrimental. For example, Yu et al.^31^ found that burn‐induced neutrophil‐secreted pro‐inflammatory cytokines TNF‐α and IL‐6 increased in a rat model with 30% total body surface area burn, promoting kidney and liver damage. Liao et al.^32^ found that neutrophil‐derived IL‐17 activated the p38 mitogen‐activated protein kinase/monocyte chemotactic protein‐1 (p38 MAPK/MCP‐1) pathway to promote ventilation‐induced lung injury. This article constructed a mouse model of sepsis and used purified mouse neutrophils for subsequent detection experiments and found that the expression of inflammatory cytokines TNF‐α, IL‐1β, and IL‐6 in activated neutrophils was significantly upregulated, consistent with previous research. Subsequent experiments further found that DOCK8 inhibited the immune function of neutrophils in sepsis. However, there are currently no reports on the regulation of DOCK8 on the immune function of neutrophils in sepsis. Therefore, we further explored the effect of DOCK8 on the immune function of neutrophils in sepsis, providing new ideas for the treatment of sepsis.

DOCK8 is an evolutionarily conserved member of the DOCK family of proteins, functioning as GEFs for the GTPase Rho family.^33^ Recent studies have shown that DOCK8 mutations can lead to a combined immunodeficiency characterized by severe and persistent viral infections, early‐onset malignancies, and atopic dermatitis.^34^, ^35^, ^36^ For example, Osnat et al.^37^ found that the representation and diversity of eukaryotic viruses were significantly increased in deep metagenomic sequencing data from DOCK8‐deficient skin samples compared to healthy volunteers. In this study, we demonstrated that DOCK8 was downregulated in neutrophils of sepsis cell and CLP mouse models and its depletion suppresses neutrophil immune function in sepsis. Further bioinformatics analysis revealed that DOCK8 was enriched in the aerobic glycolytic pathway. Metabolic reprogramming plays a central role in host defense against infection and is a novel target for inflammatory diseases.^38^ For instance, Tan et al.^39^ tested the related markers of glycolysis by constructing a mouse model of pyemia, and verified the relationship between glycolysis and pyemia by using 2‐DG as an inhibitor of glycolysis, which demonstrated that sepsis‐induced acute kidney injury can be mitigated by inhibiting aerobic glycolysis, which facilitates autophagy through the lactate/Sirtuin3/AMPK pathway. Xie et al.^40^ revealed that PKM2‐dependent glycolysis fosters the activation of NLRP3 and AIM2 inflammasomes by in vitro and in vivo assays. At present, it is generally believed that 2‐DG is an inhibitor of glycolysis, and glucose consumption and glucoamylase activity are important indicators of glycolysis function. 2‐DG treatment can significantly reduce glucose consumption and glucoamylase activity in M2 macrophages.^22^ The 2‐DG rescue test means to detect whether the level of glycolysis markers will reduce the effect of DOCK8 on glycolysis after adding 2‐DG. Here, our 2‐DG rescue assay suggested that DOCK8 may inhibit neutrophil immune function by modulating the expression of inflammatory cytokines through the regulation of aerobic glycolysis, resulting in excessive inflammation. Our findings provided new directions and targets for the study of inhibiting neutrophil immune function in sepsis.

Other studies showed that the NETs accumulation enhances in sepsis‐associated ALI patients and mice.^41^ However, our results had not been validated at the clinical levels. Meanwhile, Zhang et al.^42^ results showed that N‐acetyltransferase 10 (NAT10) is significantly downregulated in neutrophils from septic mice by regulating ULK1 RNA and activating STING pathway. But we only researched the function of DOCK8. Therefore, we plan to conduct further experiments to confirm our findings. In addition, we intend to further explore the upstream transcription factors regulating DOCK8 function to elucidate the specific mechanism by which DOCK8 inhibits sepsis immune function.

In summary, our results demonstrated that DOCK8 was downregulated in sepsis and sepsis neutrophils, suppressing neutrophil immune function in sepsis by regulating aerobic glycolysis. These findings highlighted the importance of DOCK8 in neutrophil immune function during sepsis and its potential as a therapeutic target for sepsis.

## AUTHOR CONTRIBUTIONS

**Hongjun Zhu**: Conceptualization; writing—original draft. **Junlong Xu**: Data curation. **Ke Li**: Formal analysis. **Miaomiao Chen**: Investigation. **Yueming Wu**: Methodology. **Xian Zhang**: Validation. **Hua Chen**: Data curation. **Deyuan Chen**: Writing—review & editing.

## CONFLICT OF INTEREST STATEMENT

The authors declare no conflict of interest.

## ETHICS STATEMENT

The study was approved by the Animal Care and Use Committee of Lishui University.

## Data Availability

The data that support the findings of this study are available on request from the corresponding author. The data are not publicly available due to privacy or ethical restrictions.
